# Computer-assisted screening in systematic evidence synthesis requires robust and well-evaluated stopping criteria

**DOI:** 10.1186/s13643-024-02699-7

**Published:** 2024-11-22

**Authors:** Max Callaghan, Finn Müller-Hansen, Melissa Bond, Candyce Hamel, Declan Devane, Wojciech Kusa, Alison O’Mara-Eves, Rene Spijker, Mark Stevenson, Claire Stansfield, James Thomas, Jan C. Minx

**Affiliations:** 1 Mercator Research Institute On Global Commons and Climate Change, Berlin, Germany; 2 Potsdam Institute for Climate Impact Research, Potsdam, Germany; 3 EPPI Centre, UCL Social Research Institute, University College London, London, UK; 4 Knowledge Centre for Education, University of Stavanger, Stavanger, Norway; 5 Canadian Association of Radiologists, Ottawa, ON Canada; 6 School of Epidemiology and Public Health, University of Ottawa, Ottawa, ON Canada; 7 TU Wien, Vienna, Austria; 8 Cochrane Netherlands, UMC Utrecht, Utrecht University, Utrecht, The Netherlands; 9 Medical Library, Amsterdam Public Health, University of Amsterdam, Amsterdam, The Netherlands; 10 School of Computer Science, University of Sheffield, Sheffield, UK; 11 School of Nursing and Midwifery, University of Galway, Galway, Ireland; 12 Evidence Synthesis Ireland & Cochrane Ireland, University of Galway, Galway, Ireland; 13 HRB-Trials Methodology Research Network, University of Galway, Galway, Ireland

## Introduction

Systematic reviews, systematic maps, rapid reviews, and other evidence synthesis products are important resources for evidence-based decision-making [1–3]. To synthesise evidence systematically, potentially relevant documents are usually identified using systematic searches across multiple bibliographic databases [4]. Typically, and as recommended in widely applied review guidelines, two reviewers read each of these records at the title and abstract level independently and either include for further assessment based on the full text or exclude the record [5]. This process, known as “screening”, is labour-intensive and time-consuming. As the amount of records needing to be assessed increases [6], and as progress in artificial intelligence (AI) and machine learning (ML) — particularly in the domain of text — advances [7–9], calls to use ML to increase efficiency in screening grow louder [10–12].

There is a long history of research demonstrating the potential of ML in screening (since [13]), as well as a large related literature on the use of ML for similar tasks within legal eDiscovery [14, 15]. A substantial part of this literature [16] uses ML to *prioritise* records by predicted relevance (ML-prioritised screening, sometimes referred to as active learning), such that a high proportion of potentially relevant records are identified after human screening of a lower proportion of all available records. Human screening with ML prioritisation thus constitutes an “active learning” or “researcher in the loop” procedure, in which the machine uses information from already screened documents to select which ones to show the human coders in the next batch.

Once all relevant records have been identified, the remaining unscreened records represent work that could, in principle, be saved by stopping human screening early — that is before all records have been screened. When this happens is, however, unknowable, because we do not know a priori the total number of relevant documents. To make a decision when to stop and achieve these work savings safely, a live review therefore needs methods that effectively manage the risk of missing more studies than would be acceptable in a given review. This commentary uses the term “early stopping” to refer to stopping screening before all records have been screened, without implying that this is too early. We also refer to “safe” methods for early stopping while recognising that no method eliminates risk entirely, and that the consequences of missing studies vary depending on the review context.

In this commentary, we briefly assess the current state of early stopping across evidence synthesis practice, evidence synthesis tools, and evidence synthesis guidance and highlight where this falls short of the demand for transparent and robust methods for identifying studies. In order to address this gap, we provide recommendations for promoting, developing, and applying safe stopping criteria and highlight leverage points on how to develop commonly agreed-upon principles for their implementation in ML-supported evidence synthesis.

### The state of early stopping for ML-prioritised screening

Much of the work that has followed Cohen et al. [13] has improved upon potential work savings or developed tools to allow review authors to use ML-prioritised screening. However, the field has yet to reach a consensus on when it is safe to stop screening. Many of the work savings that have been demonstrated cannot be assumed to be achievable in new reviews because those savings are based on retrospective analyses of datasets where the ideal stopping point is already known. ML-prioritised screening without early stopping can be useful, for example to frontload the identification of relevant studies so that full-text screening and data extraction can proceed in parallel or to limit double-coded records to those likely to be relevant by switching to single-reviewer screening [17]. But unlocking the considerable potential work savings that have been identified in the literature requires safe and robust methods to inform when to stop.

Without prior knowledge of the number of relevant records, any approach to stopping early risks missing relevant studies that would have been retrieved had all records been screened. Yet, to minimise the effects of publication bias and maximise the power of meta-analyses, systematic review guidelines are written to ensure that as few relevant studies are missed as possible, and that the selection process of studies is transparent, well-justified, and can, in principle, be reproduced [4]. Though traditional methods cannot entirely eliminate the risk of missing studies, the emphasis is on developing a transparent and accountable search strategy, which aims for high-recall (within practical limitations) and systematic eligibility screening of all results. Identifying studies through searching and screening relies on appropriate judgements and transparency. Where machine learning-prioritised screening is employed in systematic reviews, stopping rules should transparently manage and communicate the risk of missing relevant studies.

However, not all available stopping criteria are compatible with this requirement, partly because different criteria have been developed for different objectives and for different tasks (i.e. in legal eDiscovery). While some criteria aim to quantify the implications of stopping for recall [18, 19], others aim to trade off the costs of continued screening with the benefits of identifying additional relevant records, while others respond to the budget constraints of a project [20]. Where economic or pragmatic criteria are used to *decide* when to stop, we argue that communicating the potential consequences for recall at that point, using complementary stopping criteria, is still beneficial to support transparency and justification of the methods used.

There is limited accommodation for ML prioritisation with early stopping in the currently available systematic review guidelines. The active learning approach described in this paper has been described in Cochrane handbooks since version 6.0 [21]. Since version 6.3, the relevant section has pointed to recent literature suggesting that early stopping is not an “insurmountable” barrier. However, early stopping is not recommended, including in the current (6.4) version of the guidelines [4], and the guidelines do not help to distinguish between well-justified or poorly justified approaches to early stopping. The guidance on searching for studies published by the Campbell collaboration [22] mentions prioritised screening but only as a means to make the process efficient by frontloading relevant articles. It does not mention early stopping or stopping criteria. The PRISMA reporting guideline for systematic reviews was amended to support reporting of the increased use of ML [23], however, it only stipulates reporting the “details of automation tools used in the process”, without requiring a rationale for stopping decisions.

Several stopping criteria claim to offer safe and reliable ways to reduce work while still guaranteeing high recall [15, 18, 24–26]. Most studies evaluate how well the criterion performs on a selection of datasets by simulating the ordering of documents using ML prioritisation. However, the reliability of stopping criteria may depend on the datasets used, the ML algorithms used, their hyperparameters, or the details of how they are implemented. Reliability observed in individual evaluations may therefore be dependent on the specific experimental setup, or even be achieved by over-optimising the stopping criteria to fit the experimental setup, at the expense of generalisability. Further, evaluations of individual tools often use different metrics, making comparisons between criteria challenging. Independent and comprehensive assessments are missing. The current landscape of evaluation evidence therefore makes it hard for guideline makers to recommend safe stopping criteria.

ML-prioritised screening has been integrated into multiple citation screening platforms [17], often without the incorporation of any formal stopping rules or with the incorporation of stopping rules that have not been validated or agreed upon as good practice. Some software tools, which do not incorporate stopping rules, do not advocate early stopping [27]. Other tools leave the decision on when to stop open, while not providing guidance on which to use. Finally, some tools recommend stopping rules based on simple heuristics which are not connected to any theoretical estimate of the recall achieved [28].

The use of screening tools with ML-prioritised screening may already be widespread. According to the systematic review toolbox [29], as of January 2024, 46 digital evidence synthesis tools that focus on screening are available. Many of these incorporate ML-prioritised screening, including platforms claiming hundreds of thousands of downloads or hundreds of thousands of users [12, 27]. It is therefore likely that ML-prioritised screening is being used in numerous reviews. To safely realise the potential time savings of these tools, the community must agree on suitable rules for deciding when to stop screening.

## Recommendations

**Fig. 1:**
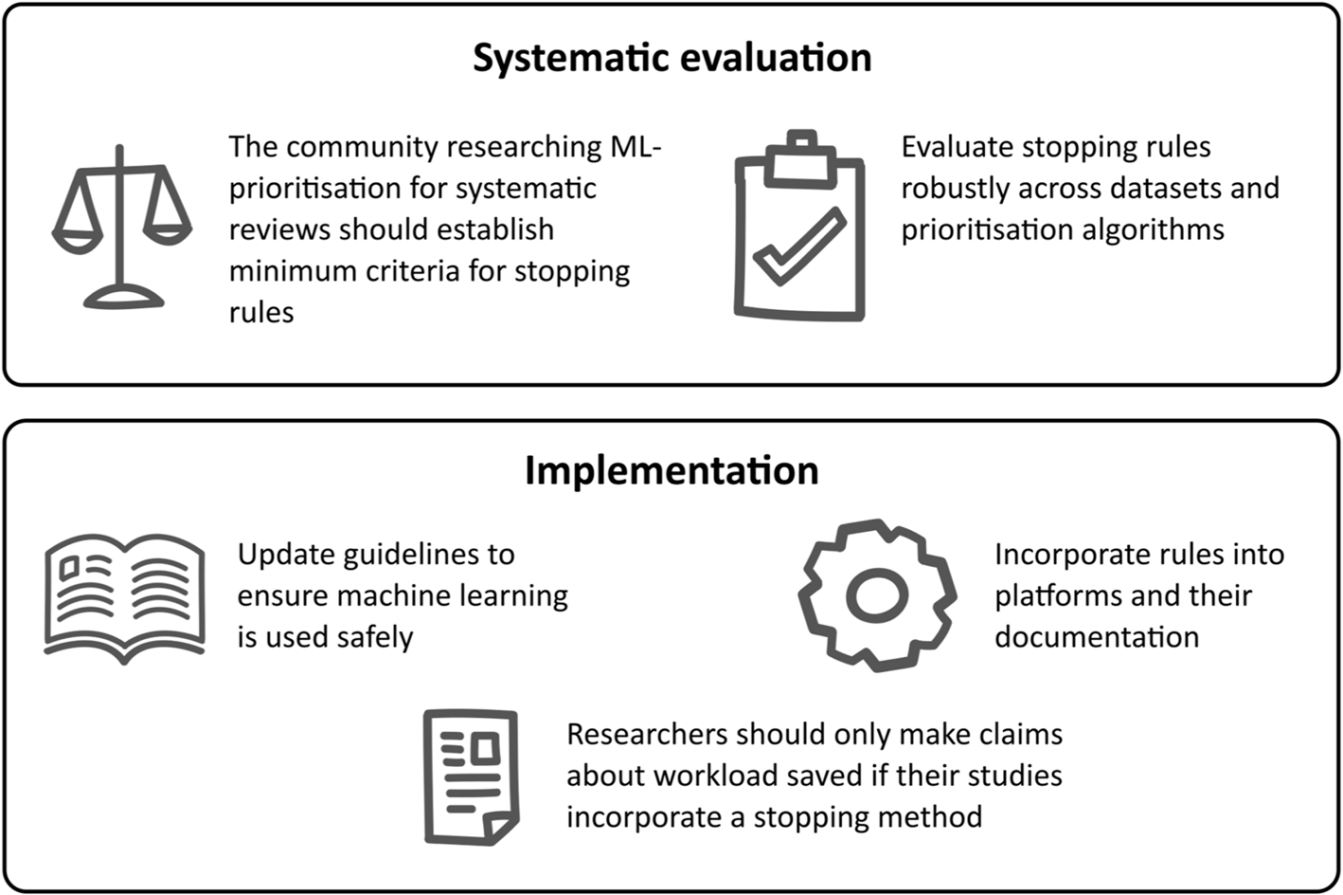
Five recommendations to advance the evaluation and implementation of safe use of stopping criteria in ML-prioritised screening

As a community of scholars and practitioners of evidence synthesis and evidence synthesis technology, including developers of evidence synthesis technology tools and authors of evidence synthesis guidelines, we have developed through discussion and debate a set of recommendations that aim to enable the successful and responsible realisation of the potential benefits of ML-prioritised screening. These recommendations (see overview in Fig. 1) respond to the issues outlined in the previous section and address the different stakeholder groups — the ML-prioritisation research community, technology developers, guideline makers, and the wider evidence synthesis community — which have a part to play in ensuring that ML can safely and responsibly be used to generate meaningful work savings in evidence synthesis screening.

### 1. The community researching ML prioritisation for systematic reviews should establish minimum criteria for stopping rules

In a systematic review context, ML-prioritised screening with early stopping aims to achieve a specific (high) level of recall while reducing workload by avoiding the need to screen all records. Recall is the ratio of relevant records identified to the total number of relevant records (at either abstract or full-text screening stage — depending on the context). However, recall’s true value is always uncertain until all records have been screened, as the total number of relevant records (i.e. the denominator in the recall equation) is unknown. Stopping rules exist to manage and communicate that uncertainty. In a gold standard systematic review, if a user is going to stop screening, they should be able to describe their confidence that a given level of predicted recall has been achieved. Only then can a reviewer assess whether the risk level is acceptable in that review’s context. In other contexts — especially in rapid reviews — reviewers may make the decision to stop screening based on economic or pragmatic considerations. However, the rationale for ceasing screening should still be communicated transparently, including an assessment of the risk of missing papers caused by stopping screening early.

Communicating the risk of missing relevant studies is possible when a stopping rule has the following attributes:

1. *It aims for a given target recall (which can be set by the user)*. Methods that tell the user when to stop without reference to a given level of recall do not allow for effective communication of the risk of missing relevant records. Moreover, different review contexts may demand different levels of recall, and a stopping rule useful for ML-prioritised screening should therefore be able to communicate the risks of missing any arbitrary level of recall.
2. *The criteria should provide explicit and reliable confidence estimates*. Stopping rules are designed to assist users in making decisions under uncertainty. They should convey a confidence level that a targeted recall level or set of levels has been achieved. Stopping rules can do this by providing a *p-score* that it is safe to stop for a given recall target or by providing a confidence interval around an estimated level of recall [15]. This confidence level should be based on sound statistical theory, stating the assumptions the calculation of the confidence interval relies upon, which should, as far as possible, be independent of the dataset and ML algorithm characteristics. Where a confidence level is stated at 95%, a stopping should be triggered before the recall target has been met 5% of the time or less, as long as the assumptions communicated hold. Criteria should be tested empirically — ideally across multiple algorithms and datasets — demonstrating that the confidence estimates are well-calibrated. Such criteria fulfil the condition of reliability defined below.
3. *Stopping criteria that aim to communicate the risk of missing studies should be free from additional parameters that require knowledge of the screening outcomes to be set effectively*. For example, stopping after X consecutive records have been excluded requires that the user sets the parameter X [30, 31]. For a specific ML-prioritised screening session in a given review, targeting a particular recall level, there will be a value of X that enables stopping the screening close to the desired recall target. However, a user has no way of knowing a priori what that value will be. Although stopping after X consecutive records may help to balance the cost of screening against the expected return of additional records, it cannot (without further analysis) reliably inform users about the potential to have missed studies. Even the more sophisticated stopping criterion implemented in SWIFT screener [19] requires that the users set a lookback parameter $\delta$. Given that this parameter decides how reliable the stopping rule is (contingent on the dataset’s attributes and the effectiveness of the ML prioritisation), the likelihood that a given recall has been achieved cannot be communicated by triggering the stopping rule at a given value of $\delta$.

### 2. Evaluate stopping rules robustly across datasets and prioritisation algorithms

Potential users of stopping criteria (and those who review and use their work) would benefit from independent and trusted guidance for understanding whether a stopping criterion is reliable or appropriate in the context of systematic reviews. This guidance should be informed by evaluations which establish, first and foremost, whether stopping criteria are safe to use and reliably avoid missing a larger number of relevant records than intended. As a secondary focus, guidance can recommend different stopping criteria based on whether they are more or less efficient (i.e. they result in as few additional records as possible that need to be screened to reach a set target recall).

#### Reliability

Reliability is a necessary condition for the use of stopping criteria to be appropriate in a gold-standard systematic review context. A stopping criterion should be considered reliable when the frequency of premature stopping aligns with the specified confidence level. For example, with a 95% confidence level, the criterion should lead to premature stopping in no more than 5% of the trials. This should hold for a large number of trials and be assessed across a large number of combinations of the following:

- Recall target
- Confidence level
- Machine learning model
- Evaluation dataset

In an ideal case, more than just being reliable on average, when the confidence level is set to 95%, a stopping rule should not be triggered too early in more than 5% of trials (plus a margin of error given by the number of trials), for any given combination of dataset, ML model, and recall target. Where this cannot be achieved, evaluations should identify the conditions under which a stopping rule cannot be relied upon. Because a user starting a new review does not know the properties of their dataset or the performance of their ML model on that dataset a priori, they should be able to trust as far as possible that the stopping rule they have chosen gives robust confidence estimates regardless of the unknowable attributes of their dataset and how their ML model will work.

#### Efficiency

To achieve a specified level of recall with a high level of confidence, any reliable stopping criteria will require that more records are screened than the minimum required to meet the recall target. This is because establishing that the recall target has been achieved under uncertainty demands extra effort. However, minimising the number of these additional records is crucial as the fewer extra records are screened, the greater are the potential work savings. The most efficient criteria are, therefore, those which require the smallest number of additional records to be screened beyond the minimum necessary to achieve the target. In a systematic review context, users should always choose a criterion that has been demonstrated to be reliable (where reliability indicates that confidence intervals are well calibrated). Where the appetite for the risk of missing studies is higher, users should adjust confidence levels, or recall targets, accordingly, rather than going for unreliable criteria with higher work savings. Among reliable criteria, users can then choose the most efficient criterion.

Assessing stopping rules across multiple combinations of recall targets, confidence levels, ML models, and datasets may also reveal that certain criteria are more efficient given particular settings. For example, stopping criterion efficiency may vary according to the combination of dataset size, prevalence or relevant records, recall target, and desired confidence level. As long as the user is selecting from among reliable criteria, users can select the criterion shown to be most efficient under the conditions of their review.

### 3. Update guidelines to ensure machine learning is used safely

The current lack of clear guidance on how to use stopping rules in screening means on the one hand that reviews which could benefit from the efficiency gains provided by ML-prioritised screening are not doing so. On the other hand, reviews are being produced using ML-prioritisation screening such that they are at risk of missing large numbers of relevant records, or not reducing their workload at all. One evaluation that ran 100 ML prioritisation runs on multiple review datasets [18] has shown that the 5th percentile of recall achieved when stopping after 50 consecutive excluded records was as low as 53%, while the most common level of work saved was 0. Such a level of risk should be considered unacceptable in any review context, and guidance should be clear that arbitrary stopping criteria have no place in high-quality systematic reviews.

Guidelines should establish minimum standards for stopping criteria, based on evaluations that show which stopping criteria are appropriate in which contexts (see “2. Evaluate stopping rules robustly across datasets and prioritisation algorithms”). They should also provide instructions on their application. For example, users and reviewers need to know what levels of uncertainty about what levels of recall are acceptable in what contexts. Having high confidence that a high level of recall has been achieved will always require that a large number of records are screened, but there may be contexts (such as in rapid reviews) where a lower level of confidence and/or a lower recall target may be acceptable. Guideline bodies should produce recommendations on the use of stopping criteria that are tailored to the specific requirements of different types of reviews.

There are further procedural questions that guidelines should inform. For example, while describing the proposed selection process in a systematic review protocol, authors should describe their proposed use of ML, including whether and how stopping rules will be used to stop early. Also, they should report in their protocol whether they pre-set their target level of recall and their confidence in achieving it or if only one of these parameters is set from the start. Depending on the resources available to conduct the review and the salience of the underlying research question, authors could also decide when to stop based on other considerations (for example resource constraints) and report the level of confidence they have in achieving a given level of recall (or range of recall levels). Guidelines should indicate which of these options are acceptable under which conditions.

Finally, guidelines should provide reporting standards to describe early stopping decisions in systematic reviews. These may stipulate that authors state their confidence level for a given recall target, or may allow for, or encourage, the reporting of confidence levels across a range of recall targets, to give a complete picture of the risks of stopping at any given point.

### 4. Incorporate stopping rules into platforms and their documentation

Many systematic review and screening platforms now incorporate ML prioritisation into their list of features. But there is considerable variation — and in some cases limited transparency — in the extent to which platforms incorporate well-justified stopping criteria that allow for well-calibrated statements about confidence that a given level of recall has been achieved. Wherever ML prioritisation is featured, it is likely that users will want to save work by stopping early, even where this is not explicitly encouraged. It is therefore incumbent on platform developers to transparently incorporate well-justified stopping criteria: to provide adequate guidance on how to use stopping criteria that are not directly incorporated into the platform or to appropriately caveat the risks of missing studies when using early stopping without robust criteria. As many users of screening platforms do not have the time, resources, or expertise to apply specific statistical tests for calculating the implications of their stopping decision, such a user-friendly implementation is paramount to wide-spread adoption of safe stopping criteria and transparent reporting about their implications.

### 5. Researchers should only make claims about workload saved if their studies incorporate a stopping method

Since the study by Cohen et al. [13], there have been dozens of papers showing how ML-prioritised screening can reduce the amount of time it takes to identify a given proportion of relevant records compared to screening the records in random order. These papers use validation datasets that have been screened manually by humans and show the point at which the target level of recall would have been achieved, had the records been screened using the ML-prioritisation approach proposed.

Unless such papers address the issue of when to stop and analyse how that approach works on their validation data, they are of limited use and have the potential to mislead. For instance, if a proposed method achieved 95% recall after screening only 40% of records in validation datasets, reviewers should not assume the same results will apply to a new review with a different dataset. A system’s performance in one context cannot guarantee similar outcomes in another. Each dataset’s unique characteristics can significantly influence the system’s effectiveness. If papers present easy-to-use software solutions for ML-prioritised screening but have missing or misleading guidance about when to stop (i.e. by using arbitrary criteria), then there is a large potential to enable a multitude of reviews which are subject to substantial risks of missing relevant studies.

Where systematic reviews use ML to prioritise screening with early stopping, authors should be challenged to justify their decision about when to stop. The use of arbitrary stopping criteria should be criticised in peer review, and authors should instead be pushed to quantify the risk that they have missed a given recall target. Only if best practices on the safe use of ML in screening are enforced in the publication process will these rules be applied and taken up by the broader evidence synthesis community, helping to reap the benefits of ML while avoiding crucial pitfalls.

## Conclusion

Systematic reviews have been developed to enable decision-making that is informed by the best available evidence. Machine learning has the potential to make screening in systematic reviews much more efficient, which is necessary in times of ever-increasing volumes of publications. However, the use of ML in prioritised screening has so far not met the same standards of methodological rigour as the rest of the systematic review process. Stopping rules are a necessary condition for ML prioritisation with early stopping to be applicable with the same rigour, but those used in practice have often been statistically incoherent and lack a sufficient basis to make decisions with such large potential to result in missing relevant studies.

Given the widespread availability of ML prioritisation in systematic review tools and platforms and the scope for such tools to be misused, guidelines must be updated to inform potential users how such tools can be used safely. The literature is now clear enough on the need for robust stopping criteria. We should apply the same standards of rigour when using ML tools as with any other part of the systematic review process.

Beyond developing robust evaluations for stopping criteria, a broader research agenda should be pursued to further enhance the safety and efficiency of using ML-prioritised screening in systematic reviews. Calibrating acceptable levels of risk for missing studies could be informed by research into the extent to which missing studies affect the results of the analysis [32, 33], as well as the extent to which increased risk of missing studies within the results of a Boolean query can be offset by enhancing other retrieval methods or even improving the query itself. Indeed, where ML-prioritised screening is not available, systematic review authors may be tempted to excessively narrow search queries in order to make screening tractable, with unquantified impacts on recall. Future research should address such trade-offs between biases introduced by compromises on search queries and potential biases introduced by ML prioritisation and only screening until a safe stopping criterion is triggered.

The interaction of stopping criteria with additional retrieval methods, as well as with decisions to single or double code records, also needs to be better understood, as does the way in which the use of ML prioritisation changes the way in which humans decide to include or exclude records [34]. For instance, stopping criteria could take into account some uncertainty in human-generated labels. The resulting higher uncertainty about the recall target could then be either reduced by double coding records for which ML predictions and human labels differ, resulting in more certainty about the human decisions, or by screening more unseen records.

Where multiple stopping criteria are shown to be reliable, the implications of combining different criteria should be evaluated. User experience could also be enhanced by research that helps to quantify the expected work savings under different conditions of the various stopping rules. Finally, more research is needed on how to apply stopping rules where new records are added in batches, such as with living systematic reviews or when an existing review is updated [35]. The systematic review community will be better served by research on practical issues of how ML-prioritised screening can be used in live reviews than in repeated demonstrations of potential work savings with retrospective analysis of fully annotated datasets.

## References

[1] Donnelly CA, Boyd I, Campbell P, Craig C, Vallance P, Walport M, Whitty CJM, Woods E, Wormald C. Four principles to make evidence synthesis more useful for policy. Nature. 2018;558(7710):361–4. 10.1038/d41586-018-05414-4. 29925978 10.1038/d41586-018-05414-4

[2] Saldanha, I. J., Adam, G. P., Schmid, C. H., Trikalinos, T. A., & Konnyu, K. J. (2023). Modernizing evidence synthesis for evidence-based medicine. In *Clinical Decision Support and beyond: Progress and Opportunities in Knowledge-Enhanced Health and Healthcare* (pp. 257–278). Elsevier. 10.1016/B978-0-323-91200-6.00006-1

[3] Surkovic E, Vigar D. Scientific advice for policymakers on climate change: the role of evidence synthesis. Philosophical Transactions of the Royal Society A: Mathematical, Physical and Engineering Sciences. 2022;380(2221):20210147. 10.1098/rsta.2021.0147. 10.1098/rsta.2021.0147 35220772

[4] Lefebvre, C., Glanville, J., Briscoe, S., Featherstone, R., Metzendorf, M.-I., Noel-Storr, A., Paynter, R., Rader, T., Thomas, J., & Wieland, L. (2023). Chapter 4: Searching for and selecting studies. In J. Higgins, J. Thomas, J. Chandler, M. Cumpston, T. Li, M. Page, & V. Welch, *Cochrane Handbook for Systematic Reviews of Interventions* (Version 6.4 (updated October 2023)). https://training.cochrane.org/handbook/current/chapter-04

[5] Higgins, J., Thomas, J., Chandler, J., Cumpston, M., Li, T., Page, M., & Welch, V. (Eds.). (2019). *Cochrane Handbook for Systematic Reviews of Interventions* (2nd ed.). John Wiley & Sons.

[6] Bornmann L, Mutz R. Growth rates of modern science: a bibliometric analysis based on the number of publications and cited references. J Am Soc Inf Sci. 2015;66(11):2215–22. 10.1002/asi.23329.

[7] Bubeck, S., Chandrasekaran, V., Eldan, R., Gehrke, J., Horvitz, E., Kamar, E., Lee, P., Lee, Y. T., Li, Y., Lundberg, S., Nori, H., Palangi, H., Ribeiro, M. T., & Zhang, Y. (2023). *Sparks of artificial general intelligence: early experiments with GPT-4* (arXiv:2303.12712). arXiv. 10.48550/arXiv.2303.12712

[8] Touvron, H., Lavril, T., Izacard, G., Martinet, X., Lachaux, M.-A., Lacroix, T., Rozière, B., Goyal, N., Hambro, E., Azhar, F., Rodriguez, A., Joulin, A., Grave, E., & Lample, G. (2023). *LLaMA: Open and Efficient Foundation Language Models* (arXiv:2302.13971). arXiv. 10.48550/arXiv.2302.13971

[9] Wolf, T., Debut, L., Sanh, V., Chaumond, J., Delangue, C., Moi, A., Cistac, P., Rault, T., Louf, R., Funtowicz, M., Davison, J., Shleifer, S., von Platen, P., Ma, C., Jernite, Y., Plu, J., Xu, C., Le Scao, T., Gugger, S., … Rush, A. (2020). Transformers: state-of-the-art natural language processing. In Q. Liu & D. Schlangen (Eds.), *Proceedings of the 2020 Conference on Empirical Methods in Natural Language Processing: System Demonstrations* (pp. 38–45). Association for Computational Linguistics. 10.18653/v1/2020.emnlp-demos.6

[10] Chappell M, Edwards M, Watkins D, Marshall C, Graziadio S. Machine learning for accelerating screening in evidence reviews. Cochrane Evidence Synthesis and Methods. 2023;1(5): e12021. 10.1002/cesm.12021. 10.1002/cesm.12021 PMC11795896 40475071

[11] Michelson M, Reuter K. The significant cost of systematic reviews and meta-analyses: a call for greater involvement of machine learning to assess the promise of clinical trials. Contemporary Clinical Trials Communications. 2019;16: 100443. 10.1016/j.conctc.2019.100443. 31497675 10.1016/j.conctc.2019.100443 PMC6722281

[12] van de Schoot, R., de Bruin, J., Schram, R., Zahedi, P., de Boer, J., Weijdema, F., Kramer, B., Huijts, M., Hoogerwerf, M., Ferdinands, G., Harkema, A., Willemsen, J., Ma, Y., Fang, Q., Hindriks, S., Tummers, L., & Oberski, D. L. (2021). An open source machine learning framework for efficient and transparent systematic reviews. *Nature Machine Intelligence*, *3*(2), Article 2. 10.1038/s42256-020-00287-7

[13] Cohen AM, Hersh WR, Peterson K, Yen P-Y. Reducing workload in systematic review preparation using automated citation classification. J Am Med Inform Assoc. 2006;13(2):206–19. 16357352 10.1197/jamia.M1929 PMC1447545

[14] Cormack, G. V., & Grossman, M. R. (2014). Evaluation of machine-learning protocols for technology-assisted review in electronic discovery. *Proceedings of the 37th International ACM SIGIR Conference on Research & Development in Information Retrieval*, 153–162. 10.1145/2600428.2609601

[15] Lewis, D. D., Gray, L., & Noel, M. (2023). Confidence sequences for evaluating one-phase technology-assisted review. *Proceedings of the Nineteenth International Conference on Artificial Intelligence and Law*, 131–140. 10.1145/3594536.3595167

[16] O’Mara-Eves A, Thomas J, McNaught J, Miwa M, Ananiadou S. Using text mining for study identification in systematic reviews: a systematic review of current approaches. Syst Rev. 2015;4(1):5. 10.1186/2046-4053-4-5. 25588314 10.1186/2046-4053-4-5 PMC4320539

[17] Hamel C, Hersi M, Kelly SE, Tricco AC, Straus S, Wells G, Pham B, Hutton B. Guidance for using artificial intelligence for title and abstract screening while conducting knowledge syntheses. BMC Med Res Methodol. 2021;21(1):285. 10.1186/s12874-021-01451-2. 34930132 10.1186/s12874-021-01451-2 PMC8686081

[18] Callaghan, M., & Müller-Hansen, F. (2020). Statistical stopping criteria for automated screening in systematic reviews. *Systematic Reviews*. 10.21203/rs.2.18218/v2 10.1186/s13643-020-01521-4 PMC7700715 33248464

[19] Howard BE, Phillips J, Tandon A, Maharana A, Elmore R, Mav D, Sedykh A, Thayer K, Merrick BA, Walker V, Rooney A, Shah RR. SWIFT-Active Screener: accelerated document screening through active learning and integrated recall estimation. Environ Int. 2020;138: 105623. 10.1016/j.envint.2020.105623. 32203803 10.1016/j.envint.2020.105623 PMC8082972

[20] Boetje J, van de Schoot R. The SAFE procedure: a practical stopping heuristic for active learning-based screening in systematic reviews and meta-analyses. Syst Rev. 2024;13(1):81. 10.1186/s13643-024-02502-7. 38429798 10.1186/s13643-024-02502-7 PMC10908130

[21] Lefebvre, C., Glanville, J., Briscoe, S., A Littlewood, Marshall, C., Metzendorf, M.-I., Noel-Storr, A., Rader, T., Shokraneh, F., Thomas, J., & Wieland, L. (2019). Chapter 4: Searching for and selecting studies. In J. Higgins, J. Thomas, J. Chandler, M. Cumpston, T. Li, M. Page, & V. Welch, *Cochrane Handbook for Systematic Reviews of Interventions* (Version 6 (updated October 2019)). https://training.cochrane.org/handbook/current/chapter-04

[22] MacDonald H, Comer C, Foster M, Labelle PR, Marsalis S, Nyhan K, Premji Z, Rogers M, Splenda R, Stansfield C, Young S. Searching for studies: a guide to information retrieval for Campbell systematic reviews. Campbell Syst Rev. 2024;20(3): e1433. 10.1002/cl2.1433. 39258215 10.1002/cl2.1433 PMC11386270

[23] Page, M. J., McKenzie, J. E., Bossuyt, P. M., Boutron, I., Hoffmann, T. C., Mulrow, C. D., Shamseer, L., Tetzlaff, J. M., Akl, E. A., Brennan, S. E., Chou, R., Glanville, J., Grimshaw, J. M., Hróbjartsson, A., Lalu, M. M., Li, T., Loder, E. W., Mayo-Wilson, E., McDonald, S., … Moher, D. (2021). The PRISMA 2020 statement: an updated guideline for reporting systematic reviews. *BMJ*, *372*, n71. 10.1136/bmj.n71 10.1136/bmj.n71 PMC8005924 33782057

[24] Molinari A, Esuli A. SALτ: efficiently stopping TAR by improving priors estimates. Data Min Knowl Disc. 2024;38(2):535–68. 10.1007/s10618-023-00961-5.

[25] Sneyd, A., & Stevenson, M. (2019). Modelling stopping criteria for search results using poisson processes. In K. Inui, J. Jiang, V. Ng, & X. W. 0001 (Eds.), *Proceedings of the 2019 conference on empirical methods in natural language processing and the 9th international joint conference on natural language processing, EMNLP-IJCNLP 2019, hong kong, china, november 3–7, 2019* (pp. 3482–3487). Association for Computational Linguistics. 10.18653/v1/D19-1351

[26] Stevenson M, Bin-Hezam R. Stopping methods for technology assisted reviews based on point processes. ACM Transactions on Information Systems. 2024;42(3):1–37. 10.1145/3631990.

[27] Walton, A. (2023, January 6). *Covidence product updates and bug fixes*. Covidence. https://www.covidence.org/blog/release-notes-december-2022-machine-learning/

[28] *How to stop screening? · asreview/asreview · Discussion #557*. (n.d.). GitHub. Retrieved 12 October 2023, from https://github.com/asreview/asreview/discussions/557

[29] *The Systematic Review Toolbox*. (n.d.). Retrieved 12 October 2023, from http://systematicreviewtools.com/software.php

[30] Jonnalagadda S, Petitti D. A new iterative method to reduce workload in systematic review process. Int J Comput Biol Drug Des. 2013;6(1–2):5–17. 10.1504/IJCBDD.2013.052198. 23428470 10.1504/IJCBDD.2013.052198 PMC3787693

[31] Przybyła P, Brockmeier AJ, Kontonatsios G, Le Pogam M-A, McNaught J, von Elm E, Nolan K, Ananiadou S. Prioritising references for systematic reviews with RobotAnalyst: a user study. Research Synthesis Methods. 2018;9(3):470–88. 10.1002/jrsm.1311. 29956486 10.1002/jrsm.1311 PMC6175382

[32] Kusa, W., Zuccon, G., Knoth, P., & Hanbury, A. (2023). Outcome-based evaluation of systematic review automation. *Proceedings of the 2023 ACM SIGIR International Conference on Theory of Information Retrieval*, 125–133. 10.1145/3578337.3605135

[33] Marshall IJ, Marshall R, Wallace BC, Brassey J, Thomas J. Rapid reviews may produce different results to systematic reviews: a meta-epidemiological study. J Clin Epidemiol. 2019;109:30–41. 10.1016/j.jclinepi.2018.12.015. 30590190 10.1016/j.jclinepi.2018.12.015 PMC6524137

[34] Scholer, F., Kelly, D., Wu, W.-C., Lee, H. S., & Webber, W. (2013). The effect of threshold priming and need for cognition on relevance calibration and assessment. *Proceedings of the 36th International ACM SIGIR Conference on Research and Development in Information Retrieval*, 623–632. 10.1145/2484028.2484090

[35] Stansfield C, Stokes G, Thomas J. Applying machine classifiers to update searches: analysis from two case studies. Research Synthesis Methods. 2022;13(1):121–33. 10.1002/jrsm.1537. 34747151 10.1002/jrsm.1537 PMC9299040

